# Influence of Boundary Conditions on Numerical Homogenization of High Performance Concrete

**DOI:** 10.3390/ma14041009

**Published:** 2021-02-20

**Authors:** Arkadiusz Denisiewicz, Mieczysław Kuczma, Krzysztof Kula, Tomasz Socha

**Affiliations:** 1 Division of Structural Mechanics, University of Zielona Góra, ul. Prof. Z. Szafrana 1, 65-246 Zielona Góra, Poland; a.denisiewicz@ib.uz.zgora.pl (A.D.); k.kula@ib.uz.zgora.pl (K.K.); t.socha@ib.uz.zgora.pl (T.S.); 2 Division of Structural Engineering, Poznan University of Technology, ul. Piotrowo 5, 60-965 Poznań, Poland

**Keywords:** boundary conditions, numerical homogenization, RVE, FEM, HPC, concrete

## Abstract

Concrete is the most widely used construction material nowadays. We are concerned with the computational modelling and laboratory testing of high-performance concrete (HPC). The idea of HPC is to enhance the functionality and sustainability of normal concrete, especially by its greater ductility as well as higher compressive, tensile, and flexural strengths. In this paper, the influence of three types (linear displacement, uniform traction, and periodic) of boundary conditions used in numerical homogenization on the calculated values of HPC properties is determined and compared with experimental data. We take into account the softening behavior of HPC due to the development of damage (micro-cracks), which finally leads to failure. The results of numerical simulations of the HPC samples were obtained by using the Abaqus package that we supplemented with our in-house finite element method (FEM) computer programs written in Python and the homogenization toolbox Homtools. This has allowed us to better account for the nonlinear response of concrete. In studying the microstructure of HPC, we considered a two-dimensional representative volume element using the finite element method. Because of the random character of the arrangement of concrete’s components, we utilized a stochastic method to generate the representative volume element (RVE) structure. Different constitutive models were used for the components of HPC: quartz sand—linear elastic, steel fibers—ideal elastic-plastic, and cement matrix—concrete damage plasticity. The numerical results obtained are compared with our own experimental data and those from the literature, and a good agreement can be observed.

## 1. Introduction

Significant progress in developing high-performance concrete (HPC) has been observed since the invention of a concrete from reactive powders (RPC) in the 1990s [1,2,3]. Concretes of this type are characterized by a much higher compressive strength, flexural tensile strength, ductility, and resilience to an aggressive environment than traditional concrete. The use of such concretes in the construction industry allows one to build challenging engineering structures (bridges and towers) and high-rising buildings. Compressive and tensile strengths and modulus of elasticity are the main mechanical parameters that affect the design properties of HPC. An accurate determination of these parameters is very important from a practical viewpoint of engineering and design for efficient and safe HPC constructions. Due to the complex composition of high-strength concrete and therefore complexity of its microstructure, which is finally formed during the hydration process, determining the abovementioned parameters is not easy [4]. Publications about the properties of cement-based composites, a name that can be also used for HPC, are concerned in most cases with experimental testing. They contain mainly parametric analysis on the influence of the volumetric fraction of individual components on the HPC response, analysis of different orientations and shape factor of fibres [5,6,7,8,9,10,11], or analysis of different types and shapes of fibres [12,13,14,15,16]. The material parameters we observe at the macro-scale are a result of phenomena taking place in the microstructure of the material, i.e. on meso-, micro-, and nano-scales. A thorough analysis of these phenomena also gives an opportunity to trace different aspects of the deformation process of HPC. One of the basic phenomena that significantly affect endurance of HPC is the formation and propagation of cracks in the microstructure of material [17,18,19].

There are many methods used to describe effective properties of heterogeneous materials, of which the most intuitive is direct homogenization [20]. It requires an estimate of the value of the averages within the area occupied by the representative volume element (RVE). The concept of RVE was defined and used by Hashin [21], where both elastic and viscoelastic properties as well as failure criteria for fibre composites where considered; by Hill [22] for elastic media; and more recently by Ostoja-Starzewski [23] in the context of plasticity of random media. The averaging operation is preceded by a solution of the boundary value problem defined for RVE with homogeneous boundary conditions. Unlike direct methods, indirect homogenization does not require estimation of the average values in the RVE cell. Indirect methods should be understood as a whole group of techniques based on Eshelby’s theory [24]. They were used in designing composite materials. Many examples of practical applications of these methods can be found in [21], including the self-consistent scheme [25], which is developed for prediction of elastic moduli of two-phase composites assuming that any (ellipsoidal) inclusion is embedded in a homogeneous medium and subject to boundary conditions at infinity. Another way to describe heterogeneity caused by the introduction of a discrete phase into the homogeneous base, i.e., matrix, is characterized by the Mori-Tanaka method [26]. Another technique to be mentioned is differential indirect method [20]. It consists of a volumetric balance matrix and discrete phase participation. A large group of homogenization methods is based on the minimum principle of potential energy, and they belong to the so-called variational homogenization methods. With their help, the lower or upper limits of parameter values are often effectively estimated [20].

Relatively recent, introduced at the turn of the 1970s and the 1980s by Bensoussan et al. [27] and Sanchez–Palencia [28], a method of asymptotic expansion for multiple scales was used [29]. It is based on a mathematical theory of homogenization, in which disturbances of unknown functions are assumed to be so small that they can be presented as an asymptotic expansion with respect to a small parameter, which is a characteristic dimension of the microstructure. This method was used to analyse materials that are physically nonlinear [30,31].

For analyzing inhomogeneous materials where the geometry and distribution of microstructure components cannot be precisely known, stochastic methods [32,33,34] can be used that also provide an estimation of properties of a surrogate material model on a macro-scale.

The last group of homogenization methods to be mentioned here are numerical methods, presented, e.g., in [35,36,37,38] and in particular for cement pastes in [39,40]. They are free from the restrictions that are imposed in strictly analytical techniques, for example, limiting the shape of a particle to the sphere or ellipse or being unable to analyze problems that are geometrically and/or physically nonlinear. In recent years, together with the increase in computing power of computers, the methods of numerical homogenization have flourished. These methods make use of computational algorithms aimed at estimating numerically the relationship between strain and stress at a macroscopic material point based on separate calculations carried out on a representative RVE that is assigned to the point and reflects the material’s microstructure there. Calculations can be carried out using various numerical techniques, of which the finite element method is the most popular one.

Modelling and computation of (high performance) concrete, owing to its complex multi-phase microstructure [5,41,42,43] and exhibiting irreversible and brittle behavior (deterioration and fracture), is an extremely difficult problem. From a computational point of view, the difficulties are manifested when solving a boundary value problem for concrete specimens or concrete structural members in the strain-softening regime, where well-posedness of the problem is lost and the pathological mesh-dependence of a solution by the finite element method occurs. In order to overcome the computational difficulties and to avoid spurious numerical results, some forms of regularization of the boundary value problem corresponding to nonlinear constitutive laws for concrete have been developed. The regularization can be achieved by incorporating into the constitutive law some nonlocal information about the deformation process from a vicinity of the material point, either by accounting for gradients of selected quantities (damage and plastic strain) or by using a phase-field approach; see Bažant et al. [44], de Borst et al. [45], Ramm et al. [46], smf Kaliske et al. [47], for example.

In this paper, we follow the procedure of a numerical two-scale homogenization approach to model the behavior of high-performance concrete, paying special attention to the boundary conditions imposed on the RVE that embodies the concrete’s microstructure. In fact, we treat the HPC and its microstructure as a composite consisting of four (or six) phases: cement matrix, fine (and thick) quartz sand, steel micro fibres (and steel fibres), and air voids. The following constitutive models were used for the components of HPC: quartz sand—linear elastic, steel fibers—ideal elastic-plastic, and cement matrix—concrete damage plasticity (CDP). Thus, the interphase and interfacial phenomena are not treated explicitly, being per assumption accounted for implicitly by the CDP model of cement matrix. In two-scale numerical homogenization, the macro-scale and micro-scale are distinguished, and at each of the scales, the corresponding boundary value problem (BVP) is solved using the finite element method. The boundary conditions imposed on the solution of the macro-scale BVP are a direct result of the support constraints of a body (structural element) and therefore are uniquely defined, whereas the boundary conditions imposed on the solution of the micro-scale BVP defined on RVE may be assumed in a number of ways. We solve the micro-scale BVP using three types of boundary conditions imposed on the boundary of RVE: uniform displacement boundary conditions (DBC), traction boundary conditions (TBC), and periodic boundary conditions (PBC).

The influence of boundary conditions used in the numerical homogenization of non-homogeneous media on the obtained values of material parameters and the stability of computational schemes have been studied by many researchers, mainly for elastic composites, e.g., in [48,49,50]. A mixed formulation of the BVP on the micro-scale was developed in [48] using weakly periodic boundary conditions, which allowed a part of microstructure to be completely cut loose by cracks and in which the idea of an elastic cohesive zone was then used as a regularization method. In order to remove rigid body motions of the elastic RVE with TBC, the concept of semi-Dirichlet boundary conditions was introduced in [49], the latter enforcing non-homogeneous displacements at selected points simultaneously satisfying the Neumann-type conditions. Numerical tests for elastic woven composites have revealed high sensitivities of the in-plane extensional modulus and Poisson’s ratio to the type of boundary conditions, and a mix of TBC and PBC proved to be best in representing an experimental strain field [50].

This contribution is a continuation of our papers [51,52,53,54], where a linear-elastic two-scale model of HPC and its experimental validation were presented. Herein, we extend our previous analyses to the case of nonlinear elasto-plastic behavior with damage. Our aim is to examine the impact of the particular type of boundary conditions on the response of two-dimensional RVE of high-performance concrete.

## 2. Materials and Methods

### 2.1. Research Methodology

The results of the numerical simulations described herein relate to the experimental findings obtained in our own laboratory tests [53] and those contained in [55]. We restrict our numerical computations to the local BVP on the RVE of high-performance concrete, checking the influence of different boundary conditions imposed on the boundary of RVE on the simulated response of the tested HPC. Accounting for randomness of the RVE microstructure, we generated positions of different phases, each in the amount as in the mix recipes in Table 1, by a pseudo-random number generator (for details, see [51]). The solution of the elasto-plastic damage BVP is solved by the finite element software package, Simulia Abaqus [56], supplemented with our in-house written script in Python and the Homtools package [57].

### 2.2. Recipes for Modelled HPC

Two mix proportions of HPC were considered, the ingredients of which are shown in Table 1. The mean compressive and tensile strengths of our two experimentally tested HPC mixtures were 106 MPa and 12.5 MPa for mixture I, and 141 MPa and 18 MPa for mixture II, cf. [53].

### 2.3. Microstructure of Modeled HPC

**Figure 1:**
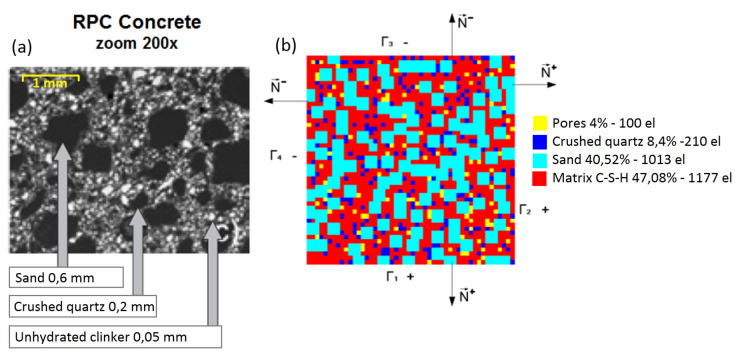
Representative volume element of high-performance concrete (HPC): (**a**) microstructure [58] and (**b**) an exemplary finite element model of representative volume element (RVE) with the given number of finite elements and wt% of components.

The microstructure of high-performance concrete has been studied by many authors, and the topic is well-recognized in the world literature, e.g., [5,41,42,43,58]. In this contribution, the microstructure of HPC is defined by means of a two-dimensional representative volume element (RVE) and the finite element method is used for modelling. The idea adopted for the numerical finite element analysis of the RVE microstructure is shown in Figure 1. The method with which we generated the considered RVE was described in [51,54]. The actual geometry of the HPC ingredients was approximated by square finite elements of dimensions 0.2 × 0.2 mm of the amount given in the concrete mix proportions table (Table 1). The size of the entire RVE was 10 × 10 mm.

**Figure 2:**
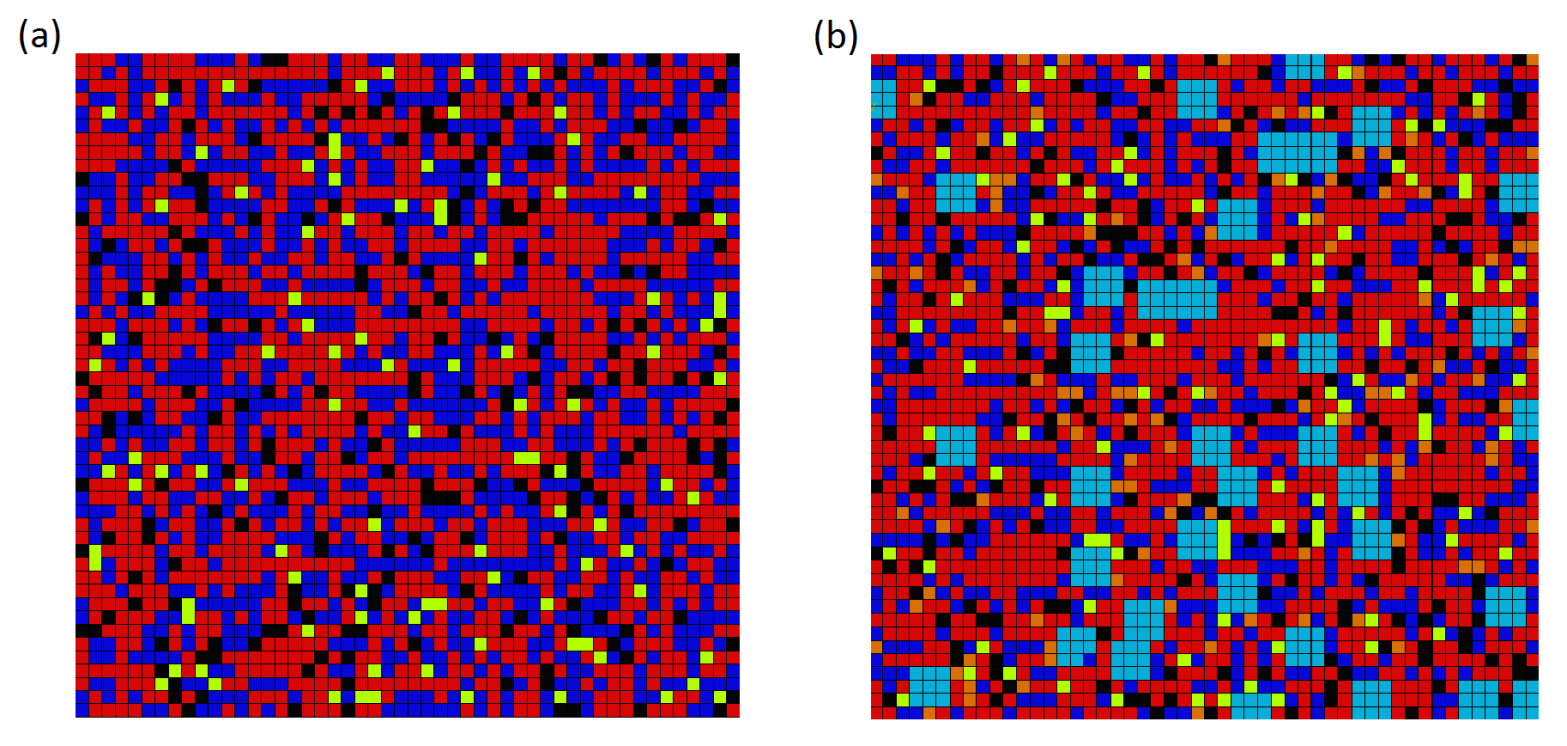
RVE for (**a**) mixture I and (**b**) mixture II. The RVE (10 × 10 mm) is divided into 2500 finite elements, each of dimensions 0.2 × 0.2 mm.

The RVE consists of several main micro-ingredients: OS 36 and OS 38-fine quartz sand, OS 30-thick quartz sand, steel (micro) fibres, and cement matrix. The constitutive models used for the components of HPC were quartz sand—linear elastic, steel fibers—ideal elastic-plastic, and cement matrix—concrete damage plasticity (Section 2.4). The RVEs of mixtures I and II were generated for numerical homogenization (Figure 2).

The components (color) of the RVE, the values of the material parameters, and wt% (densities ρ (kg/m${}^{3}$) for cement—3100, silica fume—2600, sands—2650, water and superplasticizer—1000, and steel fibres—7850) are as follows:

**Mixture I** (Figure 2a):

1. cement matrix (red): *E* = 55,000 MPa, ν = 0.17, CDP (see Section 2.4), 49.8 wt%
2. fine quartz sand (dark blue): *E* = 48,200 MPa, ν = 0.20, 37.4 wt%
3. steel micro fibres (black): *E* = 210,000 MPa, ν = 0.30, yield stress = 2100 MPa, 8.8 wt%
4. air voids (yellow): empty space (no finite elements), 4 wt%.

**Mixture II** (Figure 2b):

1. cement matrix (red): *E* = 55,000 MPa, ν = 0.17, CDP (see Section 2.4), 48.2 wt%
2. fine quartz sand (dark blue): *E* = 48,200 MPa, ν = 0.20, 22.6 wt%
3. thick quartz sand (sky blue): *E* = 73,200 MPa, ν = 0.20, 12.3 wt%
4. steel micro fibres (black): *E* = 210,000 MPa, ν = 0.30, yield stress = 2100 MPa, 8.6 wt%
5. steel fibres (orange): *E* = 210,000 MPa, ν = 0.30, yield stress = 1100 MPa, 4.3 wt%
6. air voids (yellow): empty space (no finite elements), 4 wt%.

### 2.4. Parameters of Concrete Damage Plasticity (CDP) Model

For modelling the cement-based matrix, i.e., a hardened mixture of cement, silica fume, water, and superplasticizer, we adapted the concrete damage plasticity (CDP) model as described in the influential papers [59,60,61] and Abaqus documentation [56]. Let us recall that the CDP model captures the nonlinear behavior of concrete by accounting for simultaneous development of permanent (plastic) deformation and elastic stiffness degradation using two damage variables, one for tensile damage ($d_{t}$) and the other for compressive damage ($d_{c}$), to account for different damage responses of concrete in tension and in compression. The plastic-damage model [56] assumes nonassociated potential flow with the Drucker–Prager hyperbolic function that is continuous and smooth and asymptotically approaches the linear Drucker–Prager yield condition [62]. The evolution of the elastic-plastic-damage deformation process is complex from the numerical viewpoint because it is described by inequality relations and Kuhn–Tucker complementarity conditions (loading/unloading conditions), e.g., [61,63].

The parameters of the CDP model and their values used in the calculations are gathered in Table 2, wherein

β—the internal friction angle of concrete. In the CDP model, β is defined as the inclination angle of the Drucker–Prager surface asymptote to hydrostatic axis of the meridional plane;
*m*—eccentricity of the surface of the plastic potential. This is the distance measured along the hydrostatic axis between the apex of the Drucker–Prager hyperbola and the intersection of the asymptote of this hyperbola, calculated in practice as a ratio of tensile strength to strength for compression;
$f_{b0}/f_{c0}$—number specifying the compressive strength ratio in a two-axis state for the strength in a single-axis state;
$K_{c}$—parameter defining the shape of the surface of the plastic potential on a deviatoric plane;
η—viscoplasticity parameter, used to regularize the concrete constitutive equations.

**Figure 3:**
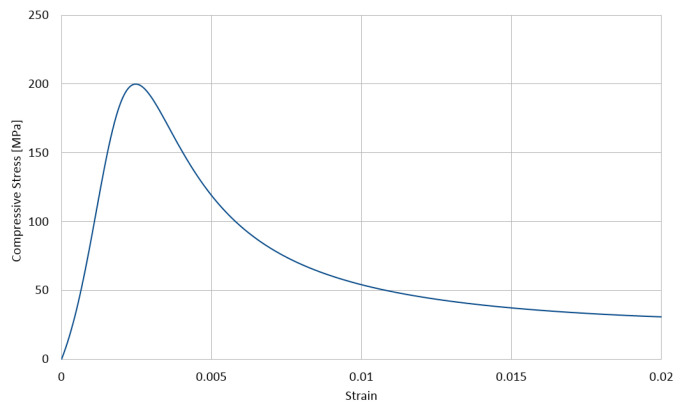
Compressive behavior of the cement matrix (concrete damage plasticity (CDP) model).

**Figure 4:**
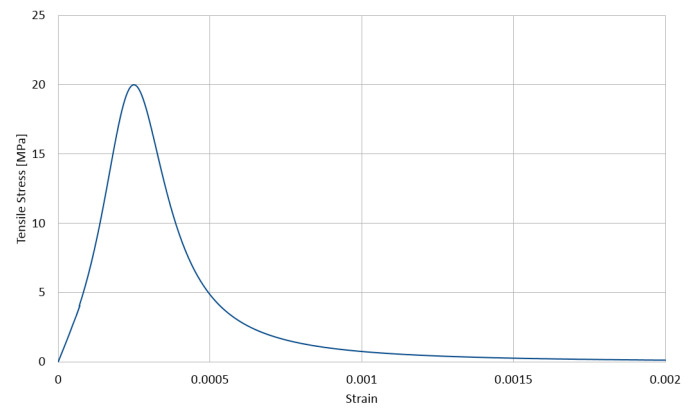
Tensile behavior of the cement matrix (CDP model).

The used stress–strain relationships for the cement matrix in compression and in tension are shown in Figure 3 and Figure 4, respectively, with the compressive strength equal to 200 MPa and the tensile strength equal to 20 MPa. These relations are described with the function proposed by Saenz [64] as

(1) $$\sigma_{\gamma }=\frac{\varepsilon_{\gamma }}{A+B\varepsilon_{\gamma }+C{\varepsilon_{\gamma }}^{2}+D{\varepsilon_{\gamma }}^{3}}$$

where

(2) $$A=\frac{1}{E},\;B=\frac{P_{3}+P_{4}-2}{P_{3}f_{\gamma m}},\;C=-\frac{2P_{4}-1}{P_{3}f_{\gamma m}\varepsilon_{\gamma 1}},\;D=\frac{P_{4}}{P_{3}f_{\gamma m}{\varepsilon_{\gamma 1}}^{2}}$$

and

(3) $$P_{1}=\frac{\varepsilon_{\gamma u}}{\varepsilon_{\gamma 1}},\;P_{2}=\frac{f_{\gamma m}}{f_{\gamma u}},\;P_{3}=\frac{E\varepsilon_{\gamma 1}}{f_{\gamma m}},\;P_{4}=\frac{P_{3}\left(P_{2}-1\right)}{{\left(P_{1}-1\right)}^{2}}-\frac{1}{P_{1}}$$

The lower index γ∈{c,t} stands for a type of stress, with *c* standing for compression and *t* standing for tension, respectively. Furthermore, $\varepsilon_{\gamma u}$ is the ultimate strain in the matrix and $f_{\gamma u}$ is its corresponding stress, whereas $f_{\gamma m}$ is the extremal stress sustained by the matrix with its corresponding strain $\varepsilon_{\gamma 1}$. In the calculations, we assumed for compression E=55 GPa, $f_{cm}=200$ MPa, $f_{cu}=196$ MPa, $\varepsilon_{cu}=0.0028$, and $\varepsilon_{c1}=0.0025$ and for tension $f_{tm}=20$ MPa, $f_{tu}=19$ MPa, $\varepsilon_{tu}=0.00028$, and $\varepsilon_{t1}=0.00025$. The values of the parameters of Equation (1) calculated by Equation (2) for compression and tension are collected in Table 3.

**Figure 5:**
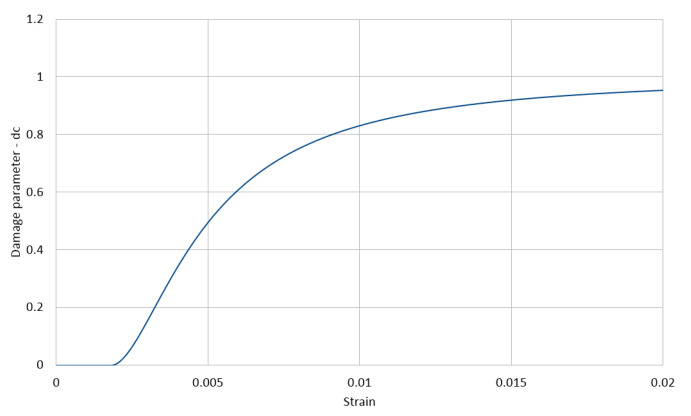
Cement matrix compression damage (CDP model).

**Figure 6:**
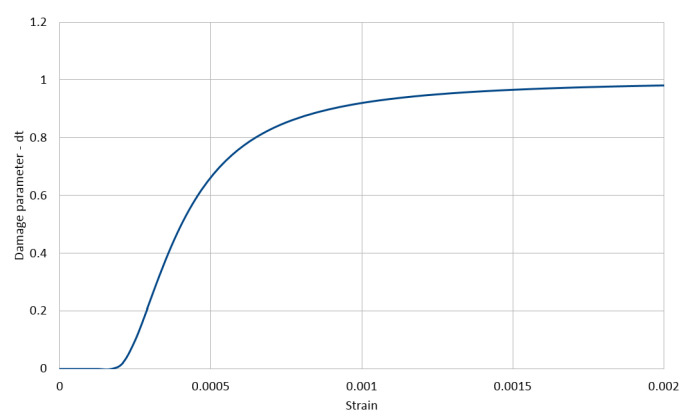
Cement matrix tension damage (CDP model).

The evolution of the compression damage scalar $d_{c}$ for the cement matrix as a function of strain and that of the tension damage scalar $d_{t}$ are illustrated in Figure 5 and Figure 6 and Equation (4), respectively.

(4) $$d_{\gamma }\left(\varepsilon_{\gamma }\right)=\frac{f_{\gamma m}-\sigma_{\gamma }\left(\varepsilon_{\gamma },\ldots \right)}{f_{\gamma m}};\;\gamma \in \left\{c,t\right\};\;d_{\gamma }\left(\varepsilon_{\gamma }\right)=0\;for\;\varepsilon_{\gamma }\leq \varepsilon_{\gamma 1}$$

### 2.5. Numerical Homogenization and Boundary Conditions

In the method of two-scale numerical homogenization, the response of a material at the macro-scale is determined by an analysis of the behavior of the material’s internal structure at the micro-scale. In the case of nonlinear material behavior, achieving the equilibrium at each scale together with the compatibility of information between the two-scales requires multiple exchanges of needed information between the scales after solving a corresponding nonlinear boundary value problems formulated at each of the scales. On the micro-scale level, the distributions of micro-stresses and micro-strains were calculated, which via homogenization provide the needed information of averaged macroscopic quantities to the macro-scale. In this contribution, our analysis was restricted to the solution of the local nonlinear BVP defined on the two-dimensional representative volume element (RVE). When the characteristic microscopic length was one order smaller than the characteristic macroscopic length, we could take into consideration only effects of the first order.

The analysis was carried out within the realm of linear kinematics. The notation that a bar above a symbol denotes a macroscopic variable or quantity was used. Let $\bar{x}$ denote the vector of coordinates of a material point at the macroscopic level, and let x stand for coordinates of points within the RVE of volume *V* defined in the material point. Further, let us assume that the microscopic displacement field u can be additively split into a linear part $\bar{\varepsilon }x$ and a fluctuating part r:

(5) $$u(\bar{x},x,t)=\bar{\varepsilon }\left(\bar{x},t\right)x+r\left(\bar{x},x,t\right)$$

where $\bar{\varepsilon }\left(\bar{x},t\right)$ is the macro-strain tensor and *t* is a time-like parameter. At down-scaling (macro-micro transition) and solving the local BVP at RVE, the strain tensor $\bar{\varepsilon }$ is treated as a known quantity (data). At up-scaling (micro-macro transition), the elements of the macro-strain tensor $\bar{\varepsilon }$ can be defined as mean values of the corresponding micro-strains ε averaged over the RVE:

(6) $$\bar{\varepsilon }=\frac{1}{\left|V\right|}\int_{V}\varepsilon \;dV=\frac{1}{\left|V\right|}\int_{\Gamma }\frac{1}{2}\left(n⊗u+u⊗n\right)\;d\Gamma$$

where n is the unit outward normal of the boundary of RVE, Γ=∂V, and ⊗ is a tensor product of vectors. The Formula (6) shows that the macroscopic strain tensor can be expressed by micro-displacements u at the boundary of the RVE. However, Equation (6) is valid provided that the zero gradient condition of the microscopic displacement fluctuation field r is satisfied:

(7) $$\int_{V}\nabla r\;dV=\int_{\Gamma }n⊗r\;d\Gamma =0$$

Fulfilling the above condition ensures that deformation of the RVE boundary in the medium sense is in accordance with the pre-set macro-strain $\bar{\varepsilon }$.

Macro-stresses can be defined, similar to macro-strains, as mean values of the micro-stresses σ:

(8) $$\bar{\sigma }=\frac{1}{\left|V\right|}\int_{V}\sigma \;dV$$

This relationship can be derived from Hill’s theorem [25], which says that the work done by macro-stresses on the corresponding macro-strains is equal to the mean value of the work performed by micro-stresses on the corresponding micro-strains:

(9) $$\bar{\sigma }\cdot \bar{\varepsilon }=\langle \sigma \cdot \varepsilon \rangle$$

where the symbol 〈•〉 stands for averaging over the RVE volume.

(10) $$\langle •\rangle =\frac{1}{\left|V\right|}\int_{V}•\;dV$$

The finite element method is applied for numerical calculations, with the finite element designated as the CPS4R Abaqus system library [56]. The RVE area was discretized with 2500 finite elements, each with dimensions of 0.2 × 0.2 mm; see Figure 2.

Numerical tests were carried out enforcing three types of boundary conditions on the boundary of RVE: linear displacement boundary conditions (DBC), uniform traction boundary conditions (TBC), and periodic boundary conditions (PBC). Before starting the numerical simulations of the inhomogeneous nonlinear BVP for high-performance concrete, the work and proper interaction between the Homtools and the Abaqus packages were verified with the example of RVE for a homogeneous material with a hole (Section 3.1).

#### 2.5.1. Linear Displacement Boundary Conditions (DBC)

This method consists of applying on the boundary of RVE the displacement field that would occur if the strain were uniform inside the RVE. For the considered linear kinematics, the boundary conditions can be defined by the formulas in Equations (5) and (7):

(11) u=〈ε(u)〉xonΓ

in which ε(u) is the micro-strain field and x is the position vector of point x on boundary Γ. There is no restriction concerning the use of this method, except that no rigid part must intersect the boundary, and holes are permitted.

#### 2.5.2. Uniform Traction Boundary Conditions (TBC)

This method consists of applying on the boundary of RVE the stress vector field that would occur if the stress were uniform inside the RVE. For the considered linear kinematics, the boundary conditions can be defined as follows:

(12) σn=〈σ〉nonΓ

where σ is the Cauchy stress tensor and n denotes the outward unit normal. There is no restriction concerning the use of this method, except that no holes must intersect the boundary.

#### 2.5.3. Periodic Boundary Conditions (PBC)

Enforcing the PBC is theoretically relevant for periodic media, which can be defined by a periodicity cell and the associated periodicity vector of translation. The periodic homogenization process consists of assuming that the strains and stresses are periodic at the level of the periodicity cell (which is defined as the RVE). The periodicity of stresses and strains leads to specific periodic boundary conditions for the localization problem on the RVE. In order to introduce periodic boundary conditions, the boundary of RVE is decomposed into two opposing parts, $\Gamma^{+}$ and $\Gamma^{-}$, such that $\Gamma =\Gamma^{+}\cup \Gamma^{-}$. Each point $x^{+}$ on $\Gamma^{+}$ is associated with a unique point $x^{-}$ on $\Gamma^{-}$, and the unit normal vectors at these boundaries satisfy $n^{-}=-n^{+}$. Then, the PBCs are defined as follows:

(13) $$u^{+}-u^{-}=\langle \varepsilon \left(x^{+}-x^{-}\right)\rangle \;\mathrm{and}\;t^{-}=-t^{+}\;\mathrm{on}\;\Gamma$$

where $t^{\pm }=\sigma n^{\pm }$ is the stress vector. For this type of boundary condition, u is forced to be periodic and t is forced to be antiperiodic. Note that LDBCs satisfy the periodicity of u only whereas TBCs satisfy the antiperiodicity of t only. There is no restriction concerning the use of this method; periodic holes and rigid parts intersecting the boundary are permitted.

## 3. Results and Discussion

### 3.1. Test Example: Homogeneous and Linear Elastic RVE with Hole

A shear test of a homogeneous linearly elastic RVE with a hole in the centre was carried out in order to check if the functioning of the Homtools package within the Abaqus calculation environment is correct. This common testing procedure preceded relevant simulations of the complex heterogeneous nonlinear BVP. It allowed us to detect the possible pitfalls in the implementation of the method of numerical homogenization. In the test, the following values of some parameters were assumed: RVE dimensions—10 × 10 mm; hole diameter—5 mm; Young’s modulus of linearly elastic material, *E* = 200 GPa; and Poisson ratio, ν = 0.3.

**Figure 7:**
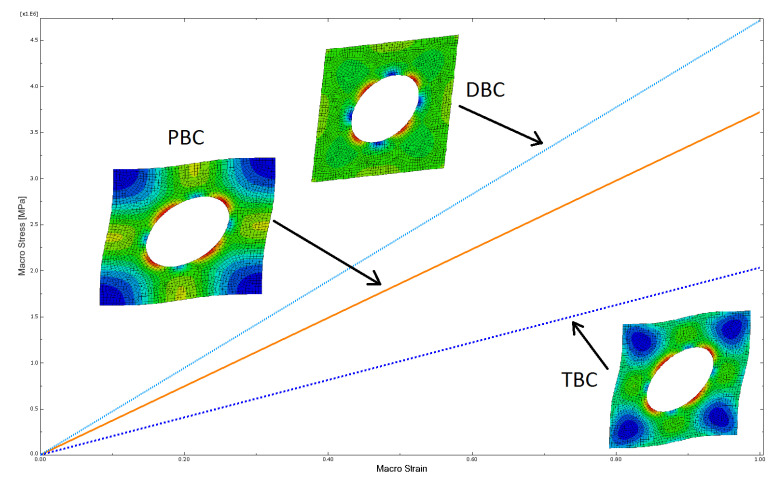
Shear test for homogeneous linear elastic RVE with a hole, demonstrating the influence of the kind of boundary condition (periodic boundary condition (PBC), displacement boundary condition (DBC), and traction boundary conditions (TBC)) on the solution.

Figure 7 shows the results of the shear test with the imposed macro-strain $\bar{\varepsilon }=\left\{0,0,1\right\}$ for different boundary conditions and the corresponding deformation modes of the RVE with the distribution of reduced micro-stresses of Huber-von Mises-Hencky (HMH). The obtained results are in good agreement with similar calculations done by other researchers, e.g., [38]. The solution of homogeneous material does not depend on the boundary condition, so the RVE without a hole was also tested. The homogeneous field of micro-stresses was obtained with the same values for the three different types of the boundary condition. It additionally confirmed the correct implementation of the used numerical homogenization scheme into the Abaqus calculation environment.

### 3.2. Compression Test for Mixtures I and II

**Figure 8:**
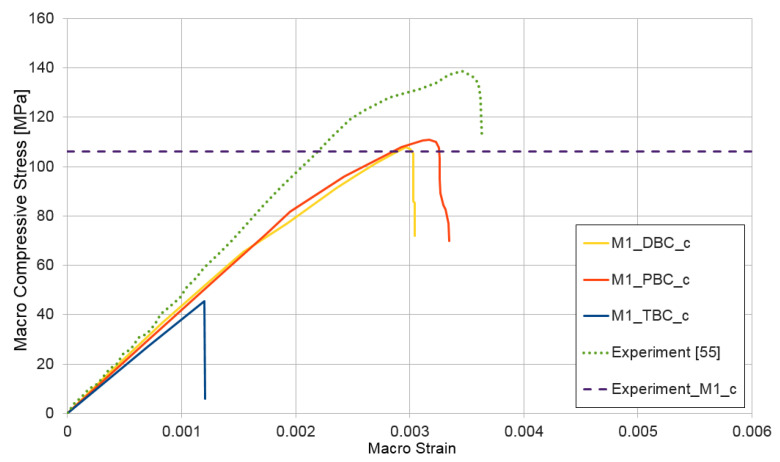
Compression test for mixture I, demonstrating the influence of the type of boundary condition (PBC, DBC, and TBC) on the solution.

A compression test $\bar{\varepsilon }=\left\{-1,0,0\right\}$ (Voigt notation) was performed for non-homogeneous RVE with nonlinear materials as a first test. The curves presented in Figure 8 show dependence between macro-strain and macro-stress for the micro-structure model of HPC (mixture I). In all presented results, the following notation was used, Mx_XXX_y, where x indicates the number of mixtures 1 or 2; XXX is the type of boundary condition: DBC, PBC, or TBC; and y is the test class (c—compression, t—tensile, and s—shear).

**Figure 9:**
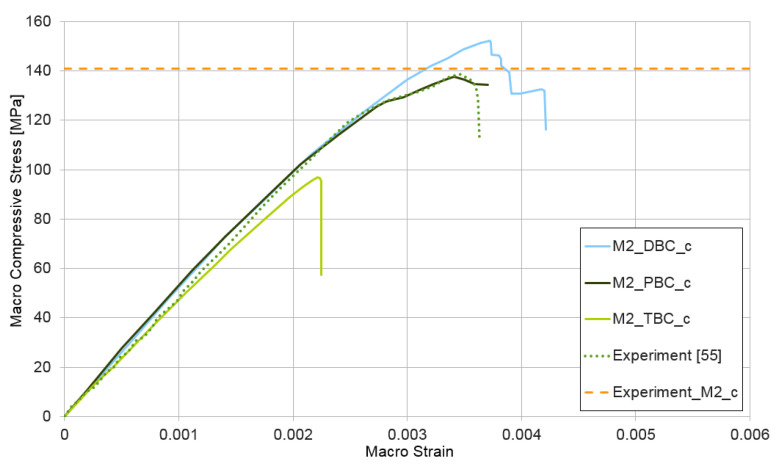
Compression test for mixture II, demonstrating the influence of the type of boundary condition (PBC, DBC, and TBC) on the solution.

**Figure 10:**
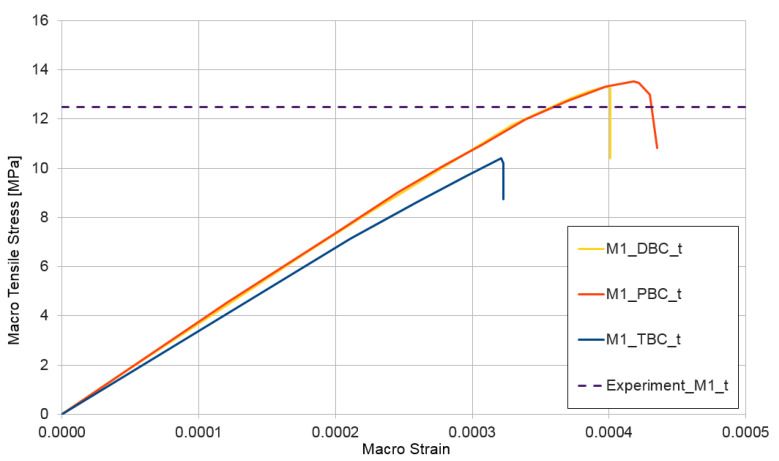
Tensile test for mixture I, demonstrating the influence of the type of boundary condition (PBC, DBC, and TBC) on the solution.

**Figure 11:**
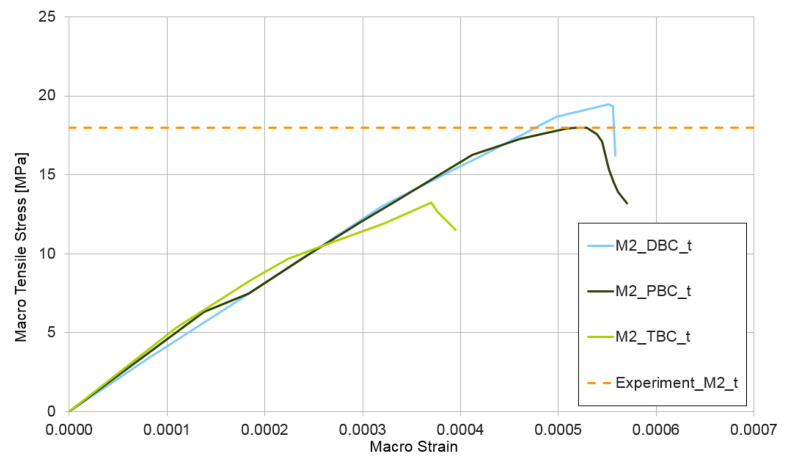
Tensile test for mixture II, demonstrating the influence of the type of boundary condition (PBC, DBC, and TBC) on the solution.

Analyzing the graphs in Figure 8, one can notice that use of the periodic boundary condition in the deformation range of RVE $\bar{\varepsilon }=\left(1.75÷3.20\right)\times 10^{-3}$ leads to an upper estimate of the compressive macro-stress. In the case of smaller deformations, the upper estimate was obtained using the displacement boundary condition. The difference between PBC and DBC is not significant; both types of conditions on the RVE boundary give similar results in the analyzed case. However, the traction boundary condition definitely leads to the lowest estimate of the macroscopic value, and under this type of boundary condition, the analyzed RVE of HPC exhibits responses that is characteristic for brittle materials—the plastic part clearly visible for the PBC and DBC does not exist for the TBC. After reaching the deformation of $\bar{\varepsilon }=1.2\times 10^{-3}$ with an imposed traction boundary condition, the rapid brittle failure of micro-structures appears. Our own experimental results were compared with the determined numerical ones to validate our computational model. In the Figure 8, the average compressive strength of six concrete specimens 100 × 100 × 100 mm made from mixture I [53] was marked (experiment_M1_c = 106 MPa). The stress–strain path could not be recorded; therefore, in Figure 8, Figure 9, Figure 10 and Figure 11, only the value of compressive or tensile strength is depicted by a dotted line. Additionally the stress–strain characteristic of concrete made by the authors of the paper [55] is presented. The recipe for that concrete is twin-like in comparison to ours for mixture I. The correlation between the experimental and the numerical results is satisfying.

A compression test $\bar{\varepsilon }=\left\{-1,0,0\right\}$ was also performed for the model of micro-structure HPC made from mixture II. The results are shown in Figure 9. In this example, the results obtained with the periodic and the displacement boundary conditions are practically identical to the deformation level of $\bar{\varepsilon }=2.22\times 10^{-3}$. The upper estimate of macroscopic values is obtained when using displacement boundary conditions in this case. Use of the traction boundary conditions also leads to an lower estimate of the macro-value. Six concrete specimens 100 × 100 × 100 mm made from mixture II [53] were also examined experimentally. The average compressive strength (marked in Figure 9 as experiment_M2_c) is equal 141 MPa, which is in very good agreement with our own numerical result, similar to the experimental results taken from the paper [55] (marked in Figure 9).

### 3.3. Tensile Test for Mixture I and II

The next test performed for the non-homogeneous RVE with material nonlinearity was a tensile test $\bar{\varepsilon }=\left\{1,0,0\right\}$. The curves presented in Figure 10 depict the obtained relation between macro-stress and macro-strain for HPC modelled as RVE-mixture I and, in Figure 11, the similar response for RVE-mixture II. Analogical to that in the compression test, the upper estimate of macro-stresses is provided for mixture I when using the periodic boundary condition and, for mixture II, when using displacement boundary condition.

The RVE deformation range in the tensile test is an order of magnitude lower than that in the compression test, which is an obvious effect of the assumed values of tensile properties for the CDP model of cement matrix. A detailed description of this phenomenon is outside the present research, but it is a very important aspect of HPC, which deserves a separate study. The authors intend to investigate the tensile response of HPC in the future. The average of the experimentally determined tensile strengths [53] is also presented in Figure 10 and Figure 11. The tensile strength was determined in the flexural test for both mixture I—experiment_M1_t = 12.5 MPa—and mixture II—experiment_M2_t = 18.0 MPa. The agreement of the experimental findings with the numerical results is very good.

**Figure 12:**
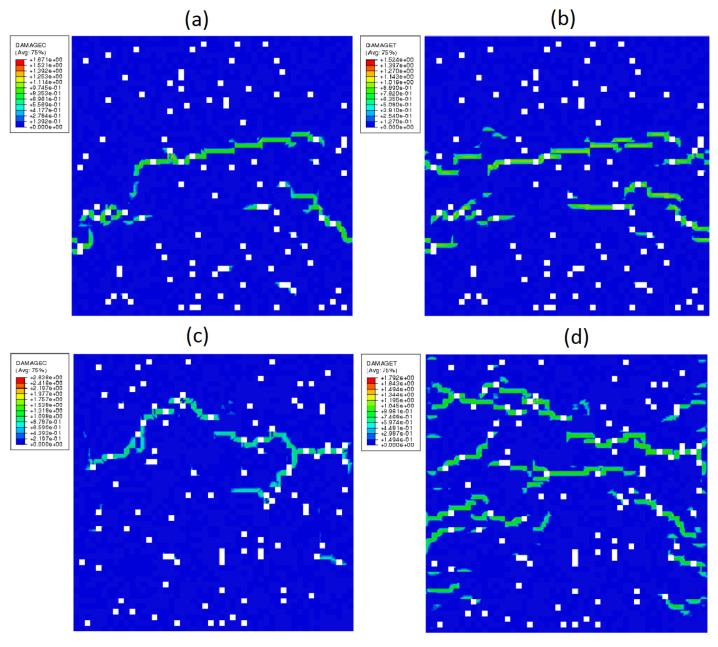
Distribution of damage parameter: (**a**) compression of mixture I with PBC; (**b**) tension of mixture I with PBC; (**c**) compression of mixture II with DBC; and (**d**) tension of mixture II with DBC. The white spots represent pores.

Figure 12 illustrates some distributions of the compressive and the tensile damage variables. The green areas indicate the places determined numerically where cracks appeared in the HCP micro-structures. A continuity of the micro-structure is breached, and a distribution of the micro-cracks is random. The latter is due to the randomly generated structure of RVE, which reflects the real conditions of HCP manufacturing. The maps of damage variable distribution shown in Figure 12 correspond to the ultimate stress (strength) sustained by the material. This value and that of the corresponding strain are for a map (a) $\bar{\sigma }=110.91$ MPa, $\bar{\varepsilon }=3.20\times 10^{-3}$; for a map (b) $\bar{\sigma }=13.50$ MPa, $\bar{\varepsilon }=4.22\times 10^{-4}$; for a map (c) $\bar{\sigma }=151.21$ MPa, $\bar{\varepsilon }=3.70\times 10^{-3}$; and for a map (d) $\bar{\sigma }=19.50$ MPa, $\bar{\varepsilon }=5.52\times 10^{-4}$.

### 3.4. Shear Test for Mixture I and II

**Figure 13:**
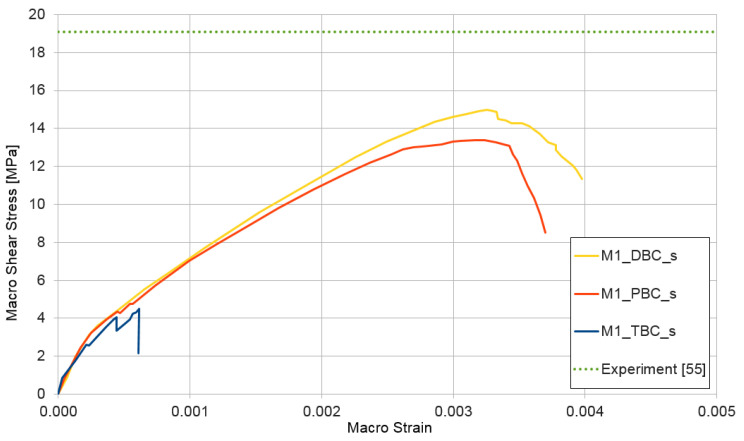
Shear test for mixture I, demonstrating the influence of the type of boundary condition (PBC, DBC, and TBC) on the solution.

**Figure 14:**
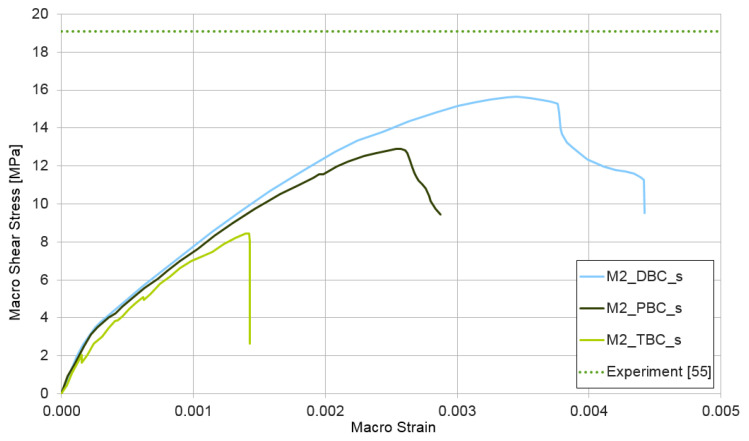
Shear test for mixture II, demonstrating the influence of the type of boundary condition (PBC, DBC, and TBC) on the solution.

The third performed numerical simulation represents a shear test $\bar{\varepsilon }=\left\{0,0,1\right\}$. The results of the test are shown in Figure 13 and Figure 14. The upper estimate of macroscopic shear stress in the shear test is provided for both cases of the RVE structure (mixture I and II) by displacement boundary conditions. The lower estimate is reached similar to that in previous cases with traction boundary conditions. The shear strength is not experimentally researched by the authors. Figure 13 and Figure 14 contain this parameter taken from [55], where the shear strength is determined for a similar concrete and on a level of 19.1 MPa, which is depicted in Figure 13 and Figure 14 by a dotted line. In this case, the difference between the experimental and the numerical results is higher by about 25% but could be accepted.

Additionally, the extreme values of the macro-stresses and the corresponding macro-strains determined in the numerical tests are collected in Table 4.

## 4. Concluding Remarks

In this paper, the technique of numerical homogenization was used for determining the macroscopic response of high-performance concrete modelled as a nonhomogeneous multi-phase nonlinear composite. The local plastic-damage boundary value problem defined on a representative volume element of the composite with complex micro-structure was solved by making use of the finite element method. Three types of boundary condition were imposed on the 2-dimensional RVE with nonlinear components: linear displacement boundary conditions (DBC), uniform traction boundary condition (TBC), and periodic boundary condition (PBC). The impact of the boundary conditions on the determined macroscopic response was studied. The obtained results show that the applied boundary conditions exert a significant influence on the calculated values of macro-quantities and that premature numerical stability problems may occur in the case of TBC.

Detailed conclusions concerning the investigated influence of a type of boundary condition on the macro-results could be summarized as follows:

- The periodic boundary conditions (PBC) lead to stable results for the full range of deformations up to failure in all performed numerical calculations.
- In the compression and tensile tests, the upper estimate of values of macro-parameters is reached for mixture I by imposing the PBC whereas for mixture II by imposing the applied displacement boundary conditions (DBC).
- Use of the DBC provides the upper estimate of values of macro-parameters in the shear test for both mixtures I and II.
- Application of the traction boundary conditions (TBC) leads in all analyzed cases to a lower estimate of values of the macro-parameters.

Validation of the numerical results by our own laboratory experiments and the experimental data from the literature shows that the presented model is capable of providing good estimations of macroscopic features for the investigated deformation process. In the first step, we restricted ourselves to an analysis of the micro-scale BVP on the RVE. In the future, it would be useful to enrich the presented analysis by using the concept of modelling error and adaptivity [65,66]. This is especially useful in the circumstance, where the behavior of a structural member as a whole is the objective and then the transition of information between the macro- and micro-scales must be performed iteratively at all integration points of the macro-finite element mesh.

## Figures and Tables

**Table 1:** Concrete mix proportions [53].

Component	Mixture I [kg/m${}^{3}$]	wt%	Mixture II [kg/m${}^{3}$]	wt%
Cement CEM I 42.5R	905	34.2	905	33.2
Silica fume	230	8.7	230	8.4
Quartz sand 0.063–0.4 mm OS 36	702	26.6	330	12.1
Quartz sand 0.04–0.125 mm OS 38	285	10.8	285	10.5
Quartz sand 0.2–0.8 mm OS 30	-	-	335	12.3
Water	260	9.8	260	9.5
Superplasticizer Woerment FM 787 BASF	29.6	1.1	29.6	1.1
Micro steel fibres DM 6/0.17 KrampeHarex	233	8.8	233	8.6
Steel fibres DW 38/1.0 N KrampeHarex	-	-	117	4.3
Density	2645	-	2725	-

**Table 2:** Mechanical parameters of the cement matrix in the plastic range.

β	*m*	$f_{b0}/f_{c0}$	$K_{c}$	η
$36^{\circ }$	0.1	1.16	0.667	0

**Table 3:** The values of the coefficients in Equation (1).

	*A*	*B*	*C*	*D*
Compression	0.00002	−0.01003	3.25030	−59.29370
Tension	0.00002	0.02236	−651.59869	1885015.6

**Table 4:** Extremal macro-stresses and corresponding macro-strains in mixtures I and II (plastic range).

Mixture_BC	Compression		Tensile		Shear	
	$\bar{\sigma }$ [MPa]	$\bar{\varepsilon }$ [-]	$\bar{\sigma }$ [MPa]	$\bar{\varepsilon }$ [-]	$\bar{\sigma }$ [MPa]	$\bar{\varepsilon }$ [-]
M1_PBC	110.91	$3.20\times 10^{-3}$	13.50	$4.22\times 10^{-4}$	13.40	$3.16\times 10^{-3}$
M1_DBC	107.18	$3.00\times 10^{-3}$	13.30	$3.98\times 10^{-4}$	14.9	$3.29\times 10^{-3}$
M1_TBC	45.55	$1.20\times 10^{-3}$	10.40	$3.21\times 10^{-4}$	4.49	$6.14\times 10^{-4}$
M2_PBC	137.77	$3.40\times 10^{-3}$	18.00	$5.29\times 10^{-4}$	12.90	$2.57\times 10^{-3}$
M2_DBC	151.21	$3.70\times 10^{-3}$	19.50	$5.52\times 10^{-4}$	15.60	$3.45\times 10^{-3}$
M2_TBC	96.61	$2.20\times 10^{-3}$	13.20	$3.70\times 10^{-4}$	8.43	$1.42\times 10^{-3}$

## Data Availability

Data sharing not available.
